# Grip Strength as a Mediator Linking Motoric Cognitive Risk Syndrome to Instrumental Activities of Daily Living

**DOI:** 10.1093/geroni/igaf122.3949

**Published:** 2025-12-31

**Authors:** Wan-Yun Chou, Su-I Hou

**Affiliations:** University of Central Florida, Orlando, Florida, United States; University of Central Florida, Orlando, Florida, United States

## Abstract

Motoric cognitive risk syndrome (MCR) represents a preclinical condition characterized by the simultaneous presence of gait impairment and cognitive complaints, typically manifesting before dementia onset. This study explores the complex mediating relationships between MCR, instrumental activities of daily living (IADL), and grip strength among Taiwanese older adults. The cross-sectional study analyzed 204 community-dwelling adults aged 65 and older who participated in annual health screenings between March 2021 and November 2022. Participants were categorized as either robust or having MCR, with MCR diagnosed when individuals scored below 1.5 standard deviations of age- and sex-matched norms on timed-up-and-go speed assessments while experiencing subjective cognitive complaints. Bootstrap analysis, with 5000 resamples, was employed to investigate how grip strength mediates the relationship between MCR and daily functioning. The prevalence of MCR reached 25% among participants. Results demonstrated a significant negative correlation between IADL score and MCR presence, with grip strength serving as a crucial mediating factor (β=-1.1055, p = 0.003). Bootstrap analysis confirmed the partial mediating effect of grip strength, explaining 33% of the total effect of MCR presence on IADL score. These findings underscore the critical importance of monitoring grip strength alongside gait speed and cognitive health to preserve daily activity function in aging populations. Understanding MCR’s underlying mechanisms could facilitate early dementia detection and prevention strategies. The research highlights the necessity of monitoring both grip strength and MCR in older adults. However, the convenience sampling methodology may limit generalizability, necessitating further investigation into grip strength’s impact on MCR-IADL relationships for broader clinical applications.

